# A conceptual health state diagram for modelling the transmission of a (re)emerging infectious respiratory disease in a human population

**DOI:** 10.1186/s12879-024-10017-8

**Published:** 2024-10-24

**Authors:** Marc Avramov, Vanessa Gabriele-Rivet, Rachael M. Milwid, Victoria Ng, Nicholas H. Ogden, Valerie Hongoh

**Affiliations:** 1 Department of Biology, Carleton University, 1125 Colonel By Drive, Ottawa, ON K1S 5B6 Canada; 2 Ottawa Research and Development Centre, Agriculture and Agri-Food Canada, 960 Carling Avenue, Ottawa, ON K1A 0C6 Canada; 3 Public Health Risk Sciences Division, Scientific Operations and Response, National Microbiology Laboratory Branch, Public Health Agency of Canada, 3200 Rue Sicotte, C.P. 5000, Saint-Hyacinthe, QC J2S 2M2 Canada; 4 Groupe de Recherche en Épidémiologie des Zoonoses et Santé Publique, Faculté de Médecine Vétérinaire, Université de Montréal, 3190 Rue Sicotte, Saint-Hyacinthe, QC J2S 2M1 Canada

**Keywords:** Mathematical modelling, Emerging disease, Reemerging disease, Pandemic, Epidemic

## Abstract

Mathematical modelling of (re)emerging infectious respiratory diseases among humans poses multiple challenges for modellers, which can arise as a result of limited data and surveillance, uncertainty in the natural history of the disease, as well as public health and individual responses to outbreaks. Here, we propose a COVID-19-inspired health state diagram (HSD) to serve as a foundational framework for conceptualising the modelling process for (re)emerging respiratory diseases, and public health responses, in the early stages of their emergence. The HSD aims to serve as a starting point for reflection on the structure and parameterisation of a transmission model to assess the impact of the (re)emerging disease and the capacity of public health interventions to control transmission. We also explore the adaptability of the HSD to different (re)emerging diseases using the characteristics of three respiratory diseases of historical public health importance. We outline key questions to contemplate when applying and adapting this HSD to (re)emerging infectious diseases and provide reflections on adapting the framework for public health-related interventions.

## Background

One of the challenges arising from the recent COVID-19 pandemic was the need for the rapid development of mathematical models, many of which were developed within academic institutions, some in collaboration with public health organisations. The evolution of knowledge regarding the epidemiology and biology of COVID-19 necessitated the simultaneous adjustment of the models to produce realistic and relevant results (e.g. contribution of asymptomatic transmission). This required ongoing collaboration with subject matter experts to appropriately modify models based on the disease biology, as well as the public health measures that were implemented to control transmission [1–5]. Without biological reality, modelling can produce results that can range across a spectrum of accuracy from good to inaccurate which may lead to criticism, justified or not, of modelling as a source of support for public health decisions [6].

When an infectious disease (re)emerges in a population, the first priority for scenario-type modelling is to estimate the potential burden (e.g. number of cases, hospitalisations, and deaths) on the population and assess the effect of control measures that can be used to reduce the impact of the disease. To guide the development of (re)emerging infectious disease models that are realistic from both biological and public health perspectives, we developed a health state diagram (HSD) to allow mapping of sequential infection and disease states as the causal pathogen spreads in the population. The HSD applies to both compartmental and agent-based model types and allows the assessment of the impacts of public health measures on transmission to control an epidemic. It lays out the processes involved in pathogen transmission and progression of infection that are common to respiratory infections and allows the incorporation of public health responses that can be explored in such scenarios. Consequently, when used with disease- and intervention-specific components, the HSD can act as a guide for modellers on model construction, not only in terms of model development but also to allow experts with whom they may need to collaborate with to adequately parameterise the models as scientific evidence grows [5]. In this article, we explore the utility of the COVID-19-inspired HSD both as a starting point for reflecting on evolving knowledge of a (re)emerging respiratory pathogen and in evaluating the feasibility of potential control measures. We also discuss important considerations in refining the HSD as knowledge and control measures evolve.

### Proposed health state diagram and initial considerations

**Fig. 1:**
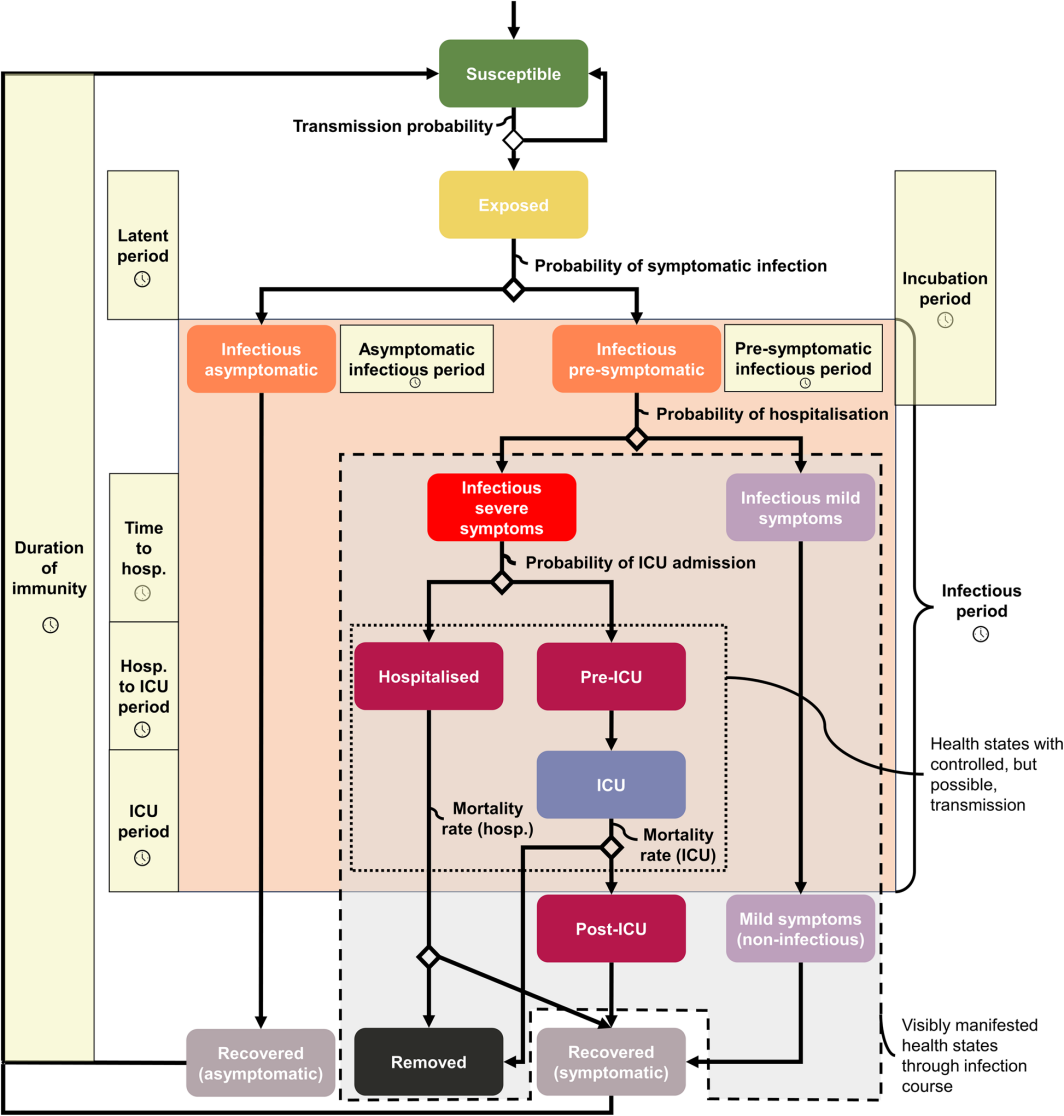
Health state diagram for respiratory infectious disease modelling. A health state diagram with 14 states: an initial *susceptible* state prior to exposure to infection, an *exposed* state once successful transmission occurs, a *pre-symptomatic* state where an individual is infected but not yet symptomatic, multiple forms of infectious states ranging from *asymptomatic*, *mild* (including *moderate*), *severe*, *hospitalised*, requiring intensive care unit (*ICU*) care, followed by post-infection states including *recovered* (and potentially immune) or *dead* individuals (and thus removed). Disease state transition times (denoted by the temporal parameters) are disease-specific. The dashed box depicts the states where disease occurrences are typically identified within the population (e.g. symptomatic and hospitalised states). The hospitalised and ICU states enclosed within the dotted-line box may be infectious but are assumed to be isolated and thus not contributing to community transmission in this HSD

At the onset of the COVID-19 pandemic, the Public Health Agency of Canada developed several models including compartment and agent-based models, to evaluate the impact of nonpharmaceutical interventions (NPIs) on epidemic progression [1, 2]. First inspired by a generic influenza transmission model [7], the COVID-19 agent-based model rapidly evolved to include additional disease states as their importance became apparent (e.g. the pre- and asymptomatic states and varying severities of disease manifestation by age group) as well as approximation of age-structured population mixing in representative households, mixed-age venues, and schools using contact matrices [1, 2, 8]. The COVID-19 pandemic provided invaluable insights into the utility of mathematical modelling and highlighted the need for readily adaptable disease models. Transmission models used at the beginning of the pandemic were simple (e.g. Susceptible-Exposed-Infectious-Recovered model), when the importance of additional key states was not yet known. Here we propose a slightly more complex HSD (Fig. 1) that captures the early transmission dynamics of a (re)emerging disease. The HSD is based on the first year of the COVID-19 pandemic, before vaccination became available, and comprises the states of infection that are common for almost every infectious disease. As a disease (re)emerges in a population, it spreads through *susceptible* individuals. *Exposed* individuals – those that had effective contact with *infected* individuals – become infectious following a latent period and may develop symptoms (*symptomatic*) following an incubation period (depending on the disease, the latent period may be shorter or the same duration as the incubation period). *Infected* individuals may experience *symptomatic* or *asymptomatic* forms of infection. *Symptomatic* cases can range from *mild* symptoms (e.g. in the case of a flu, runny nose) to more severe symptoms requiring *hospitalisation*. *Mild* cases (here, encompasses both mild and moderate cases, but combined into a single state for simplicity) recover on their own, while severely symptomatic cases will require hospitalisation with the most severe requiring treatment in intensive care units (*ICU*). The outcomes of more severe cases can be either *recovery* or *death*. In diseases without life-long immunity, a return to susceptibility may follow after a certain length of time recovered. Most, if not all, of these states occur for all respiratory infectious diseases, but diseases differ in the details – the rate of infection, the duration of the latent, asymptomatic, symptomatic, and infectious periods, the proportion of asymptomatic infections, the infection-hospitalisation, infection-ICU utilisation and infection-fatality rates, and details of the strength and duration of post-infection immunity.

The HSD delineates both visible and non-visible states that can occur as disease transmission unfolds in a population. Observable (visible) states serve as empirical benchmarks aiding in model validation and parameter inference, where simulations can be aligned with data [9, 10]. Conversely, non-visible states, such as asymptomatic and pre-symptomatic states, have not always been included in past models, perhaps due to a presumed negligible contribution to transmission dynamics or considered non-relevant to the modelling objective [11]. We suggest early consideration of non-visible states when constructing new models for a (re)emerging disease to encourage reflection on where public health interventions should be applied for effective containment [12–15].

While symptomatic states are generally visible early on during a pandemic, severe cases are more likely to be detected, especially in hospital settings, isolated and their contacts traced and quarantined by public health organisations [16]. The proportions of severe manifestations (actual and model-predicted) are essential for public health experts in determining the potential impact on the public and healthcare capacity. Higher severity, or virulence, may mean a greater impact on healthcare, but when coupled with lower transmissibility may also mean that a larger proportion of cases are detected, isolated and for whom contacts are traced [17]. Early estimation at local, regional, or national scales, of the proportion of individuals, by age or vulnerability status, likely to develop severe outcomes and necessitate access to healthcare infrastructure, enables models to better project healthcare needs for both resource planning and decisions on public health measures. In the case of COVID-19, early studies from cases in Asia, and China in particular, were instrumental in estimating key parameters used for modelling [17–19].

Waning of immunity, the existence of which was not confirmed in the early phase of the COVID-19 pandemic, is included in our proposed HSD given the importance this played as the pandemic persisted even after vaccines became available [20–22]. Its potential existence in a newly (re)emerging disease should be considered in terms of available public health control measures and assessing the potential effectiveness these can have in controlling an outbreak over time.

Although some public health measures may seem irrelevant for certain modelling objectives, their inclusion should still be considered as they will have an impact on pathogen transmission dynamics (Table 1). Non-pharmaceutical public health measures can include case detection and isolation, contact tracing and quarantine, masking and physical distancing and, in extreme cases, lockdowns. Depending on test sensitivity and the strength of surveillance mechanisms in place, case detection may occur at different stages within the HSD. Case detection can enable isolation, contact tracing and quarantine of potentially infected contacts. Measures to reduce the probability of transmission to susceptible individuals upon contact with an infectious individual, such as physical distancing and masking, can be implemented on individuals in various states within the HSD. Pharmaceutical interventions include vaccines and therapeutics. Vaccines (which are unlikely to be available at the start of a pandemic) may help in reducing the probability of infection, developing symptoms, or developing severe symptoms, depending on the nature of the vaccine, and may therefore impact individuals in multiple states throughout the HSD. Therapeutics such as antivirals (if available and effective), which aim to limit the severity of infections, may have a window of time during which they can be effectively used and thus specific states to which they can be applied.

**Table 1:** Potential public health measures available for control of (re)emerging infectious diseases

Public health measure	Description
Case detection	Identifies infected individuals often via clinician diagnosis and/or laboratory diagnostic test. Depending on the disease and available tests, case detection generally occurs once symptoms appear, (e.g. starting from *infectious mild* or *infectious severe symptomatic* states) but with an extensive surveillance system and appropriate tests, case detection can occur before symptoms present (e.g. *infectious pre-symptomatic* and *infectious asymptomatic* states) [23]. Case detection can be followed by isolating infected individuals to prevent further transmission (next public health measure).
Isolation	Prevents the spread of the disease from detected cases to *susceptible* individuals. It implicates any infectious health state by isolating individuals who can transmit the disease, yet do not need hospitalisation. Occurs following diagnosis [24].
Contact tracing	Identifies contacts of known cases. It implicates individuals of any stage between the *exposed* and *infectious* states, but ideally before they become infectious (i.e., while in the *exposed* state, or *susceptible* state when no effective contact was ultimately made [25]. Contact tracing is followed by quarantining of contacts to prevent further transmission (next public health measure).
Quarantine	Isolates individuals who were identified as a contact (i.e. may have been exposed to an infected person). It implicates any stage between the *susceptible* and *infectious* states, by isolating *susceptible* individuals or *infectious* individuals who can or will potentially transmit the disease [24]. Generally occurs following contact tracing.
Masking	Reduces the likelihood of transmission from *infectious* individuals (*infectious* individuals spreading infectious droplets) and the likelihood of *susceptible* individuals becoming infected (*susceptible* individuals inhaling infectious droplets). Similar to physical distancing, masks can affect all *infectious* states and can be a protective intervention for the *susceptible* state [26].
Physical distancing	Reduces the likelihood of close contact between *susceptible* and *infectious* individuals by maintaining a safe distance from one another. Mostly applies to *infectious* states, but also applies to *susceptible* individuals by providing some protection against effective contact with an *infectious* individual. Can be implemented through modifications in the contact rates for all individuals in the model [27].
Shutdowns (i.e. lockdowns)	Restrictions to limit movement of agents to specific environments, thus reducing contact between potentially *infectious* and *susceptible* individuals and hence disease spread. Lockdowns can involve the closure of non-essential businesses, schools, and public spaces, as well as restrictions on gatherings and travel [28].
Treatment	Provides appropriate care based on the severity of the illness, therefore influencing the outcomes and duration of illness for individuals in any of the severe symptom states. To prevent further complications, especially for more vulnerable groups, some treatments are provided without the need for hospitalisation [29].
Vaccination	Stimulates the immune systems of susceptible individuals and provides protection from specific disease outcomes if exposed and/or infected. Depending on the type of vaccine, it may affect all health states throughout the entire course of infection [30].

For each public health measure, we describe the general mechanism in which the measures can affect the course of infection in the health state diagram

#### Applicability to diverse infectious diseases

To illustrate the framework’s utility, we explored its application to three respiratory viruses of historical public health importance, including measles, SARS-CoV-1, and influenza. These diseases have historical relevance in Canada, given their rapid evolution into pandemics (e.g. the 2009 H1N1 pandemic), and have sociodemographic implications associated with the vulnerability of the primary groups affected (e.g. measles and children or influenza and the elderly). These diseases are situated at three different extremes of severity and transmissibility, and we explore the HSD’s flexibility across these ranges. We emphasise the need for reflection on the range of possible health states across disease transmission and progression to contribute to more informed public health control planning.

#### Measles

Measles, a vaccine-preventable disease, is notable for its highly infectious nature and distinctive rash development, particularly in young children [31–33]. Measles vaccination is generally thought to be lifelong, though studies suggest immunity wanes over time [34, 35]. A recent resurgence in outbreaks among non-immunized individuals in countries previously free of the disease is becoming an increasing concern due in part to COVID-19 related interruptions to childhood vaccination schedules [31–33]. As vaccination for measles exists, its inclusion or some proxy (e.g. proportion of the population assumed to be already immune), would be necessary to capture present-day outbreak dynamics, but otherwise, the proposed HSD could be used to model measles transmission in a population.

Significant transmission may occur while only mild, non-distinct respiratory virus symptoms are being experienced, up to four days before the indicative rash development which follows an 8- to 12-day incubation period [36]. Asymptomatic states have not frequently been incorporated into measles models [37, 38], though evidence suggests that these exist and can contribute to transmission among susceptible individuals [38–40]. Variations in symptoms range from mild, generally for those with partial pre-existing immunity, to more severe forms that can involve multiple organ systems and generally necessitate hospital care with a potential for death [36, 41]. Severity of symptoms varies by age with children under five years of age particularly vulnerable [36, 41].

#### SARS-CoV-1

The 2003 SARS-CoV-1 pandemic was notably distinct from COVID-19 due to its initial presentation with more severe symptoms. High infection fatality rates occurred [42]; however, epidemics in countries where it emerged were relatively short-lived, in part because the virus was only transmissible once symptoms appeared, which were more severe and more quickly identified. In turn, control measures, such as case isolation and tracing and quarantine of contacts, were more easily and successfully applied [11].

Examining the HSD for use with SARS-CoV-1 shows general applicability. During the 2003 pandemic, there was little to no reported asymptomatic transmission and few cases with mild symptoms [43]. The effective use of quarantine of both asymptomatic contacts and mild cases successfully contained spread [43]. A majority of reported cases necessitated hospital treatment [43]. Nosocomial transmission occurred, but cases were rapidly contained via isolation [44]. Waning immunity of SARS-CoV-1 infection was fortunately not tested due to the successful eradication of SARS-CoV-1 infections in 2003.

#### Influenza

The burden from pandemic versus seasonal forms of influenza generally differs due to existing immunity in the population and the age of affected vulnerable groups (i.e. children versus elderly). Both forms of influenza, with the exception of the 1918 influenza, have generally been less virulent and transmissible than COVID-19, SARS-CoV-1 and measles. Pre-symptomatic and asymptomatic transmission is known to occur, though perhaps due to the perceived tolerance of mild symptoms, control measures are generally focused on reducing the burden of the most severe cases through vaccination [11, 45]. A brief examination of the literature shows good support for the HSD with influenza given the variation in the severity of symptoms and existence of non-symptomatic states reported. More explicit tracking and accounting of these health states might elicit different perspectives on effective public health control options for influenza.

#### Further considerations for (re)emerging diseases

Modelling generally aims to help test our understanding of disease transmission, ascertain disease burden, and assess the potential effectiveness of control measures. The proposed HSD, delineating general disease progression through potential visible and non-visible states, leaves room for adapting the model depending on the stage of the epidemic and modelling objective at hand.

The effectiveness of a given control method depends on its attributes and the transmission and virulence characteristics of the causal pathogen. In the application of the HSD to a (re)emerging disease, a fundamental question to address is the primary purpose of the modelling exercise, which is likely to change depending on the stage of the outbreak. Defining the specific modelling objectives will guide the adaptation of the HSD to meet the desired outcomes.

First, once early estimates of transmission parameters and age-related infection, hospitalisation, and fatality rates become available, early-stage modelling can assess the possible impact of an uncontrolled epidemic, and thus the urgency of implementing control. The HSD framework can be used to help think through the existing understanding of epidemic progression throughout the population. Questions to ask during this stage are whether evidence exists for the inclusion or omission of all proposed stages in the HSD. Where uncertainty exists, can a range of estimates be used to see how these states might affect the transmission dynamics, or are certain states irrelevant to the modelling objective and thus can these be safely omitted? Second, modelling can estimate the capacity of NPIs to control the epidemic given different levels of effort, often to maintain incidence at levels that healthcare can cope with, to avoid overwhelming situations as seen in Italy early in the COVID-19 pandemic [46]. Questions such as: where do NPIs effectively intersect with the HSD and how does their inclusion impact transmission? and, can states irrelevant to the modelling objective (e.g. if asymptomatic individuals are found not to contribute to transmission), be safely omitted without leading to severe under- or over-estimation of the effectiveness of potential NPIs?. Third, if and when a vaccine has been developed, and information on vaccine performance at limiting severe outcomes and transmission, becomes available, modelling can estimate the necessary levels of vaccine uptake by the population to allow lifting of NPIs. Here, it is important to ask whether the vaccine is severity-limiting or transmission-limiting and how this will impact disease progression. In the context of waning immunity, the introduction of new variants of concern for a disease may also impose modifications of modelling parameters [47].

Additional questions to consider are, whether the (re)emerging disease exhibits variations in severity based on socioeconomic and/or demographic factors, and whether these should be incorporated into the model to help understand the impact of the disease on different population groups. This includes examining factors such as age, income, co-morbidities (including immunodeficiencies), or other vulnerabilities that may influence disease transmission and outcomes. Although out of scope of this article, further consideration of human behaviour and how it might impact transmission, for example through levels of compliance to recommended public health measures, should be considered when modelling. Finally, where applicable, the impact of potential environmental transmission (e.g. via contaminated surfaces) should be carefully considered in modelling parameterisation.

It is important to note that numerous framework-based opinions, reviews, and perspectives have been proposed in the far and recent past for modelling transmission dynamics (e.g. [3, 9, 48–50]). The HSD and items discussed in this article are not intended to be argued as definitive solutions to the inherent complexities of mathematical modelling of respiratory pathogen transmission. Instead, we set out to aid with the thought exercises that accompany the framing of modelling objectives, which is especially key when informed public health decisions are needed early in the (re)emergence of a pathogen in the population.

## Conclusion

With important hindsight from the COVID-19 pandemic, the HSD serves as a starting point, easily adaptable by activating or deactivating the aforementioned aspects as necessitated by disease dynamics and/or the evolving comprehension of epidemics/pandemics. The adaptability of this framework is an asset in scenarios involving (re)emerging infectious diseases. In the event of a novel pathogen, the HSD provides an initial comparative reference of how infectious diseases generally progress through a population. Within the context of epidemic and pandemic response strategies, it allows rapid evaluation of possible intervention strategies even in the absence of extensive disease-specific data.

The HSD offers a valuable conceptual framework; however, its practical application necessitates ongoing refinement and validation through empirical data. Continuous data collection of parameters specific to individual diseases remains imperative to enhance the accuracy and reliability of transmission dynamics modelling. Its potential to expedite response planning, provide a springboard for novel pathogen assessments, and offer standardised modelling approaches underscores its value as a foundational tool for mitigating (re)emerging infectious disease threats.

## Data Availability

No datasets were generated or analysed during the current study.

## Abbreviations

HSD: Health State Diagram
NPI: Nonpharmaceutical intervention
ICU: Intensive care unit

## References

[1] Ng V, Fazil A, Waddell LA, Bancej C, Turgeon P, Otten A, et al. Projected effects of nonpharmaceutical public health interventions to prevent resurgence of SARS-CoV-2 transmission in Canada. Can Med Assoc J. 2020;192(37):E1053-64. 32778573 10.1503/cmaj.200990 PMC7513947

[2] Ludwig A, Berthiaume P, Orpana H, Nadeau C, Diasparra M, Barnes J, et al. Assessing the impact of varying levels of case detection and contact tracing on COVID-19 transmission in Canada during lifting of restrictive closures using a dynamic compartmental model. Can Commun Dis Rep. 2020;46(1112):409–21. 33447163 10.14745/ccdr.v46i1112a08 PMC7799879

[3] Sherratt K, Carnegie AC, Kucharski A, Cori A, Pearson CAB, Jarvis CI, et al. Improving modelling for epidemic responses: reflections from members of the UK infectious disease modelling community on their experiences during the COVID-19 pandemic. Wellcome Open Res. 2024;9:12. 38784437 10.12688/wellcomeopenres.19601.1 PMC11112301

[4] Medley GF. A consensus of evidence: the role of SPI-M-O in the UK COVID-19 response. Adv Biol Regul. 2022;86:100918. 36210298 10.1016/j.jbior.2022.100918 PMC9525209

[5] Corrin T, Ayache D, Baumeister A, Young K, Pussegoda K, Ahmad R, et al. COVID-19 literature surveillance—a framework to manage the literature and support evidence-based decision-making on a rapidly evolving public health topic. Can Commun Dis Rep. 2023;49(1):5–9. 36815866 10.14745/ccdr.v49i01a02 PMC9902036

[6] Ioannidis JPA, Cripps S, Tanner MA. Forecasting for COVID-19 has failed. Int J Forecast. 2022;38(2):423–38. 32863495 10.1016/j.ijforecast.2020.08.004 PMC7447267

[7] Brauer F, Castillo-Chavez C, Feng Z. Models for influenza. In: Mathematical models in epidemiology. New York: Springer New York; 2019. p. 311–50. (Texts in Applied Mathematics; vol. 69). Available from: http://link.springer.com/10.1007/978-1-4939-9828-9_9. Cited 2024 Mar 11.

[8] Prem K, Cook AR, Jit M. Projecting social contact matrices in 152 countries using contact surveys and demographic data . Halloran B, editor. PLOS Comput Biol. 2017;13(9):e1005697. 28898249 10.1371/journal.pcbi.1005697 PMC5609774

[9] Jia N, Tsui L. Epidemic modelling using Sars as a case study. North Am Actuar J. 2005;9(4):28–42.

[10] MacIntyre CR. The discrepant epidemiology of Middle East respiratory syndrome coronavirus (MERS-CoV). Environ Syst Decis. 2014;34(3):383–90. 32288979 10.1007/s10669-014-9506-5 PMC7104603

[11] Fraser C, Riley S, Anderson RM, Ferguson NM. Factors that make an infectious disease outbreak controllable. Proc Natl Acad Sci. 2004;101(16):6146–51. 15071187 10.1073/pnas.0307506101 PMC395937

[12] Montgomery MP, Morris SE, Rolfes MA, Kittikraisak W, Samuels AM, Biggerstaff M, et al. The role of asymptomatic infections in influenza transmission: what do we really know. Lancet Infect Dis. 2023;24(6):e394–e404. 10.1016/S1473-3099(23)00619-9. 10.1016/S1473-3099(23)00619-9 PMC11127787 38128563

[13] Regoes RR, Bonhoeffer S. Emergence of drug-resistant influenza virus: population dynamical considerations. Science. 2006;312(5772):389–91. 16627735 10.1126/science.1122947

[14] Stilianakis NI, Perelson AS, Hayden FG. Emergence of drug resistance during an influenza epidemic: insights from a mathematical model. J Infect Dis. 1998;177(4):863–73. 9534957 10.1086/515246

[15] Asplin P, Keeling MJ, Mancy R, Hill EM. Epidemiological and health economic implications of symptom propagation in respiratory pathogens: a mathematical modelling investigation. Lam TTY, editor. PLOS Comput Biol. 2024;20(5):e1012096. 38701066 10.1371/journal.pcbi.1012096 PMC11095726

[16] Xiang Y, Jia Y, Chen L, Guo L, Shu B, Long E. COVID-19 epidemic prediction and the impact of public health interventions: a review of COVID-19 epidemic models. Infect Dis Model. 2021;6:324–42. 33437897 10.1016/j.idm.2021.01.001 PMC7790451

[17] Russell TW, Golding N, Hellewell J, Abbott S, Wright L, Pearson CAB, et al. Reconstructing the early global dynamics of under-ascertained COVID-19 cases and infections. BMC Med. 2020;18(1):332. 33087179 10.1186/s12916-020-01790-9 PMC7577796

[18] Lauer SA, Grantz KH, Bi Q, Jones FK, Zheng Q, Meredith HR, et al. The incubation period of coronavirus disease 2019 (COVID-19) from publicly reported confirmed cases: estimation and application. Ann Intern Med. 2020;172(9):577–82. 32150748 10.7326/M20-0504 PMC7081172

[19] Kucharski AJ, Russell TW, Diamond C, Liu Y, Edmunds J, Funk S, et al. Early dynamics of transmission and control of COVID-19: a mathematical modelling study. Lancet Infect Dis. 2020;20(5):553–8. 32171059 10.1016/S1473-3099(20)30144-4 PMC7158569

[20] Hernandez-Suarez C, Murillo-Zamora E. Waning immunity to SARS-CoV-2 following vaccination or infection. Front Med. 2022;9: 972083. 10.3389/fmed.2022.972083 PMC9606629 36313998

[21] Saad-Roy CM, Morris SE, Boots M, Baker RE, Lewis BL, Farrar J, et al. Impact of waning immunity against SARS-CoV-2 severity exacerbated by vaccine hesitancy. Wallqvist A, editor. PLOS Comput Biol. 2024;20(8):e1012211. 39102402 10.1371/journal.pcbi.1012211 PMC11299835

[22] Angelov G, Kovacevic R, Stilianakis NI, Veliov VM. An immuno-epidemiological model with waning immunity after infection or vaccination. J Math Biol. 2024;88(6). Available from: https://link.springer.com/10.1007/s00285-024-02090-z. Cited 2024 Sep 17. 10.1007/s00285-024-02090-z PMC11052727 38668894

[23] Wee LE, Fua T, Chua YY, Ho AFW, Sim XYJ, Conceicao EP, et al. Containing COVID-19 in the emergency department: the role of improved case detection and segregation of suspect cases. Kline JA, editor. Acad Emerg Med. 2020;27(5):379–87. 32281231 10.1111/acem.13984 PMC7262126

[24] Khan MA, Atangana A, Alzahrani E, Fatmawati. The dynamics of COVID-19 with quarantined and isolation. Adv Differ Equ. 2020;2020(1):425. 32834821 10.1186/s13662-020-02882-9 PMC7427274

[25] Tupper P, Otto SP, Colijn C. Fundamental limitations of contact tracing for COVID-19. Pai N, editor. FACETS. 2021;6:1993–2001.

[26] Gurbaxani BM, Hill AN, Patel P. Unpacking Cochrane’s update on masks and COVID-19. Am J Public Health. 2023;113(10):1074–8. 37672741 10.2105/AJPH.2023.307377 PMC10484132

[27] Li L, Taeihagh A, Tan SY. A scoping review of the impacts of COVID-19 physical distancing measures on vulnerable population groups. Nat Commun. 2023;14(1):599. 36737447 10.1038/s41467-023-36267-9 PMC9897623

[28] Caulkins JP, Grass D, Feichtinger G, Hartl RF, Kort PM, Prskawetz A, et al. The optimal lockdown intensity for COVID-19. J Math Econ. 2021;93: 102489. 33558783 10.1016/j.jmateco.2021.102489 PMC7857053

[29] Cascella M, Rajnik M, Aleem A, Dulebohn SC, Di Napoli R. Features, evaluation, and treatment of coronavirus (COVID-19). In: StatPearls. Treasure Island (FL): StatPearls Publishing; 2024. Available from: http://www.ncbi.nlm.nih.gov/books/NBK554776/. Cited 2024 Apr 15. 32150360

[30] Coccia M. Optimal levels of vaccination to reduce COVID-19 infected individuals and deaths: a global analysis. Environ Res. 2022;204: 112314. 34736923 10.1016/j.envres.2021.112314 PMC8560189

[31] Sanyaolu A, Okorie C, Marinkovic A, Ayodele O, Abbasi AF, Prakash S, et al. Measles outbreak in unvaccinated and partially vaccinated children and adults in the United States and Canada (2018–2019): a narrative review of cases. Inquiry. 2019;56:004695801989409. 10.1177/0046958019894098 PMC6906342 31823676

[32] Georgakopoulou T, Horefti E, Vernardaki A, Pogka V, Gkolfinopoulou K, Triantafyllou E, et al. Ongoing measles outbreak in Greece related to the recent european-wide epidemic. Epidemiol Infect. 2018;146(13):1692–8. 30086813 10.1017/S0950268818002170 PMC9507959

[33] Patel M, Lee AD, Clemmons NS, Redd SB, Poser S, Blog D, et al. National update on measles cases and outbreaks — United States, January 1–October 1, 2019. MMWR Morb Mortal Wkly Rep. 2019;68(40):893–6. 31600181 10.15585/mmwr.mm6840e2 PMC6788396

[34] Yang L, Grenfell BT, Mina MJ. Waning immunity and re-emergence of measles and mumps in the vaccine era. Curr Opin Virol. 2020;40:48–54. 32634672 10.1016/j.coviro.2020.05.009

[35] He H, Chen E, fu, Li Q, Wang Z, Yan R, Fu J, et al. Waning immunity to measles in young adults and booster effects of revaccination in secondary school students. Vaccine. 2013;31(3):533–7. 23159458 10.1016/j.vaccine.2012.11.014

[36] Leung AK, Hon K, Leong K, Sergi C. Measles: a disease often forgotten but not gone. Hong Kong Med J. 2018;24(5):512. 30245481 10.12809/hkmj187470

[37] McLean HQ, Fiebelkorn AP, Temte JL, Wallace GS, Centers for Disease Control and Prevention. Prevention of measles, rubella, congenital rubella syndrome, and mumps, 2013: summary recommendations of the Advisory Committee on Immunization Practices (ACIP). MMWR Recomm Rep. 2013;62(RR–04):1–34. 23760231

[38] Sutcliffe PA, Rea E. Outbreak of measles in a highly vaccinated secondary school population. CMAJ. 1996;155(10):1407–13. 8943928 PMC1335111

[39] Vardas E. Isolation of measles virus from a naturally-immune, asymptomatically re-infected individual. J Clin Virol. 1999;13(3):173–9. 10443793 10.1016/s1386-6532(99)00026-8

[40] Riley EC, Murphy G, Riley RL. Airborne spread of measles in a suburban elementary school. Am J Epidemiol. 1978;107(5):421–32. 665658 10.1093/oxfordjournals.aje.a112560

[41] Perry RT, Halsey NA. The clinical significance of measles: a review. Orenstein WA, editor. J Infect Dis. 2004;189(Supplement_1):S4-16. 15106083 10.1086/377712

[42] Jia N, Feng D, Fang L, Richardus JH, Han X, Cao W, et al. Case fatality of SARS in mainland China and associated risk factors. Trop Med Int Health. 2009;14(s1):21–7. 19508439 10.1111/j.1365-3156.2008.02147.x PMC7169690

[43] Hui DSC, Chan MCH, Wu AK, Ng PC. Severe acute respiratory syndrome (SARS): epidemiology and clinical features. Postgrad Med J. 2004;80(945):373–81. 15254300 10.1136/pgmj.2004.020263 PMC1743054

[44] Chan-Yeung M, Xu R. SARS: epidemiology. Respirology. 2003;8:s1. 10.1046/j.1440-1843.2003.00518.x PMC7169193 15018127

[45] Ip DKM, Lau LLH, Leung NHL, Fang VJ, Chan KH, Chu DKW, et al. Viral shedding and transmission potential of asymptomatic and pauci-symptomatic influenza virus infections in the community. Clin Infect Dis. 2017;64(6):736–42. 10.1093/cid/ciw841 PMC5967351 28011603

[46] Indolfi C, Spaccarotella C. The outbreak of COVID-19 in Italy. JACC Case Rep. 2020;2(9):1414–8. 32835287 10.1016/j.jaccas.2020.03.012 PMC7270641

[47] Otto SP, Day T, Arino J, Colijn C, Dushoff J, Li M, et al. The origins and potential future of SARS-CoV-2 variants of concern in the evolving COVID-19 pandemic. Curr Biol. 2021;31(14):R918-29. 34314723 10.1016/j.cub.2021.06.049 PMC8220957

[48] Klepac P, Metcalf CJE, McLean AR, Hampson K. Towards the endgame and beyond: complexities and challenges for the elimination of infectious diseases. Philos Trans R Soc B Biol Sci. 2013;368(1623):20120137. 10.1098/rstb.2012.0137 PMC3720036 23798686

[49] Heesterbeek H, Anderson RM, Andreasen V, Bansal S, De Angelis D, Dye C, et al. Modeling infectious disease dynamics in the complex landscape of global health. Science. 2015;347(6227). Available from: https://www.science.org/doi/10.1126/science.aaa4339. Cited 2024 Sept 16. 10.1126/science.aaa4339 PMC4445966 25766240

[50] Grenfell BT, Pybus OG, Gog JR, Wood JLN, Daly JM, Mumford JA, et al. Unifying the epidemiological and evolutionary dynamics of pathogens. Science. 2004;303(5656):327–32. 14726583 10.1126/science.1090727

